# BurkDiff: A Real-Time PCR Allelic Discrimination Assay for *Burkholderia Pseudomallei* and *B. mallei*

**DOI:** 10.1371/journal.pone.0015413

**Published:** 2010-11-12

**Authors:** Jolene R. Bowers, David M. Engelthaler, Jennifer L. Ginther, Talima Pearson, Sharon J. Peacock, Apichai Tuanyok, David M. Wagner, Bart J. Currie, Paul S. Keim

**Affiliations:** 1 Translational Genomics Research Institute, Flagstaff, Arizona, United States of America; 2 Northern Arizona University, Flagstaff, Arizona, United States of America; 3 Mahidol-Oxford Tropical Medicine Research Unit, Faculty of Tropical Medicine, Mahidol University, Bangkok, Thailand; 4 Menzies School of Health Research, Darwin, Australia; National Institutes of Health, United States of America

## Abstract

A real-time PCR assay, BurkDiff, was designed to target a unique conserved region in the *B. pseudomallei* and *B. mallei* genomes containing a SNP that differentiates the two species. Sensitivity and specificity were assessed by screening BurkDiff across 469 isolates of *B. pseudomallei*, 49 isolates of *B. mallei*, and 390 isolates of clinically relevant non-target species. Concordance of results with traditional speciation methods and no cross-reactivity to non-target species show BurkDiff is a robust, highly validated assay for the detection and differentiation of *B. pseudomallei* and *B. mallei*.

## Introduction

The bacterial species *Burkholderia pseudomallei* and *B. mallei*, though genetically very similar, have divergent lifestyles. *B. pseudomallei* is a soil saprophyte and facultative pathogen and the cause of melioidosis, while *B. mallei* is an obligate pathogen and the cause of glanders. Melioidosis is mostly a disease of humans and animals in Southeast Asia and Northern Australia, where *B. pseudomallei* is present in the environment; infection mainly results from percutaneous inoculation or inhalation or aspiration of the organism. Clinical manifestations of melioidosis can be asymptomatic, localized to virtually any organ, or disseminated, though the primary presentations are pneumonia and sepsis, where mortality rates are significant [1], [2]. Glanders is mainly an equine disease found in much of the world, except for North America, Europe and Australia, with transmission to humans occurring primarily through direct contact with animals and aerosols [3], [4]. Clinical manifestations of glanders in humans are similar to those of melioidosis [1], [5]. Both species of bacteria cause fast-progressing diseases and both are intrinsically resistant to several antibiotics. As such, the rapid detection and identification of these species is essential for immediate appropriate patient therapy. Both species are also potential bioterrorism agents, deemed by the U.S. Centers for Disease Control and Prevention Category B Select Agents [6], for which no human vaccine is available. In this context, rapid differentiation of melioidosis and human glanders is paramount for epidemiological surveillance and forensic investigation.

Identification of *B. pseudomallei* and *B. mallei* and the diagnosis of melioidosis and glanders currently depend on time-consuming culture of the organism. Confirmation by biochemical assays can add a week onto definitive species identification [1]. Rapid biochemical assays have resulted in misdiagnosis of melioidosis, a mistake not easily detected due to the myriad clinical manifestations of the disease, and the lack of vigilance for these organisms in non-endemic regions [7], [8]. Serologic assays can be erroneous [8], are contingent on a delayed immune response, and are useful really only in non-endemic areas, where seroconversion due to previous exposure is improbable [1]. Antigen-specific assays, including direct immunofluorescent microscopy [9] and latex agglutination [10], have proven to be rapid and sensitive, but are not as yet available commercially.

Molecular methods to identify *B. pseudomallei* and *B. mallei* now exist that utilize various platforms: Sanger sequencing [11], multiplex PCR [12], real-time PCR [13], [14], [15], [16], and isothermal DNA amplification [17]. Several of these assays show promise as rapid alternatives to biochemical tests; however few have been extensively validated for robustness and specificity. *B. pseudomallei* and *B. mallei* are relatively genetically promiscuous, making development of robust, specific single-locus assay diagnostics challenging [18], [19].

A single-reaction real-time PCR Taqman allelic discrimination assay was previously developed to identify and differentiate *B. pseudomallei* and *B. mallei* [20]. Further analysis of this assay against larger strain collections revealed some false positive identification: a strain of *B. oklahomensis* types as *B. pseudomallei*, and the *B. thailandensis*-like strain MSMB43 types as *B. mallei* (unpublished data).

Here we introduce a new more highly validated allelic discrimination assay, referred to as BurkDiff, to provide a higher level of specificity for accurate identification of *B. pseudomallei* and *B. mallei* and simultaneous differentiation when necessary. Alternatively, as these two species often occupy disparate niches under normal circumstances, BurkDiff can be used as a single-probe assay for definitive identification of *B. pseudomallei* or *B. mallei*.

## Methods

We used the methods described by Pearson et al. [21] to compare 23 *B. pseudomallei* and 10 *B. mallei* genomes to search for shared orthologous SNPs, then filtered them by mismatch value (the distance to the next SNP in bases). We further analyzed the resultant pool of SNPs and their flanking regions with a GenBank BLAST search, and finally chose one for assay development and validation.

Using Primer Express 3.0 software (Life Technologies, Foster City, CA), we designed a Taqman SNP dual-probe allelic discrimination assay in which one probe was designed to hybridize with the *B. mallei* allele (5′-FAM-CTGAAACGCGCAGCG-3′-MGB) and the other to the *B. pseudomallei* allele (5′-VIC-CTGAAACGCGAAGCG-3′-MGB). Real-time PCR was carried out in 10 uL reactions containing 900 nM of both forward (5′-CGAGCGCATCGTACTCGTA-3′) and reverse (5′- CAAGTCGTGGATGCGCATTA-3′) primers, 200 nM of each probe, 1X Applied Biosystems Genotyping Mastermix, and 0.5 ng template. Thermal cycling and endpoint analysis was performed on an AB 7900HT sequence detection system (Life Technologies) using the following conditions: 50°C for 2 min, 95°C for 10 min, and 40 cycles of 95°C for 15 s and 58°C for 1 min.

To evaluate the utility of this SNP and its locus as a diagnostic marker for *B. pseudomallei* and *B. mallei*, we used the Taqman allelic discrimination assay to genotype a collection of human, animal, and environmental isolates of *B. pseudomallei* (n = 469) and *B. mallei* (n = 49) from a broad geographic range (Table 1). Additionally we assessed specificity by screening isolates of near-neighbor species (n = 62), and isolates of species of similar clinical presentation or normal flora (n = 328) (Table 2). All isolates were originally identified by standard microbiological techniques in the laboratory of origin.

**Table 1:** Number and origin of *B. pseudomallei*, *B. mallei*, and genetic near-neighbor strains used in this study.

Species	Country	Isolated from	No. of isolates	TaqMan result (SNP state)
*B. mallei*	China	Human	2	C
	China	Animal	4	C
	China	Unknown	2	C
	France	Unknown	1	C
	Hungary	Animal	1	C
	Hungary	Unknown	1	C
	India	Animal	3	C
	India	Unknown	1	C
	Pakistan	Unknown	6	C
	Turkey	Human	4	C
	Turkey	Animal	1	C
	Turkey	Unknown	10	C
	UK	Unknown	1	C
	USA	Human	4	C
	USA	Animal	1	C
	USA	Unknown	3	C
	Unknown	Animal	2	C
	Unknown	Unknown	2	C
Total	8		49	
*B. pseudomallei*	Australia	Human	131	A
	Australia	Animal	10	A
	Australia	Environmental	57	A
	Australia	Unknown	6	A
	Bangladesh	Human	2	A
	Cambodia	Unknown	2	A
	China	Unknown	3	A
	Ecuador	Human	2	A
	Ecuador	Animal	1	A
	Fiji	Human	1	A
	India	Unknown	1	A
	Indonesia	Environmental	1	A
	Kenya	Human	1	A
	Kenya	Environmental	2	A
	Laos	Unknown	2	A
	Madagascar	Environmental	2	A
	Malaysia	Human	2	A
	Malaysia	Environmental	3	A
	Malaysia	Unknown	15	A
	Mauritius	Human	1	A
	Pakistan	Human	2	A
	Papua New Guinea	Human	1	A
	Papua New Guinea	Unknown	1	A
	Puerto Rico	Human	2	A
	Singapore	Human	2	A
	Singapore	Environmental	1	A
	Sweden	Human	1	A
	Thailand	Human	89	A
	Thailand	Environmental	105	A
	Unknown	Human	1	A
	Unknown	Environmental	2	A
	Unknown	Unknown	2	A
	USA	Human	6	A
	Venezuela	Human	1	A
	Vietnam	Human	4	A
	Vietnam	Animal	1	A
	Vietnam	Unknown	3	A
Total	22		469	
*B. cepacia*	USA		2	Negative
*B. oklahomensis*	USA		2	Negative
*B. thailandensis*			58	Negative
Total			62	

**Table 2:** Species and number of differential diagnostic and background flora strains screened across BurkDiff to validate the assay's specificity.

Species	No. of strains	Species	No. of strains
*Abiotrophia/Granulicatella* grp	1	*Neisseria gonorrhoeae*	4
*Achromobacter xylosoxidans*	1	*Neisseria meningitidis*	3
*Acinetobacter baumanni*	7	*Pasteurella multocida*	1
*Bacillus anthracis*	1	*Propionibacterium sp.*	1
*Bacillus cereus*	1	*Providencia stuartii*	1
*Bacillus sp.*	2	*Pseudomonas aeruginosa*	7
*Bacteroides fragilis*	1	*Rhizopus oryzae*	1
*Bacteroides uniformis*	1	*Rothia mucilaginosa*	1
*Bordetella bronchiseptica*	1	*Salmonella enterica*	1
*Brucella abortus*	1	*Shigella dysenteriae*	1
*Brucella suis*	1	*Staphylococcus arlettae*	1
*Candida albicans*	5	*Staphylococcus aureus*	55
*Candida glabrata*	2	*Staphylococcus capitis*	1
*Candida parapsilosis*	3	*Staphylococcus cohnii*	1
*Candida tropicalis*	1	*Staphylococcus epidermidis*	8
*Chryseobacterium indologenes*	1	*Staphylococcus equorum*	1
Coagulase negative *Staphylococcus*	16	*Staphylococcus gallinarum*	1
*Coccidioides immitis*	1	*Staphylococcus haemolyticus*	3
*Coccidioides posadasii*	2	*Staphylococcus hominis*	1
*Corynebacterium diphtheriae*	1	*Staphylococcus kloosii*	1
*Corynebacterium jeikeium*	1	*Staphylococcus lugdunensis*	1
*Coxiella burnetii*	2	*Staphylococcus saprophyticus*	2
*Enterobacter aerogenes*	2	*Staphylococcus xylosus*	3
*Enterobacter cloacae*	10	*Stenotrophomonas maltophilia*	1
*Enterococcus faecalis*	9	*Streptococcus agalactiae*	9
*Enterococcus faecium*	6	*Streptococcus anginosus*	2
*Escherichia coli*	11	*Streptococcus equi*	1
*Francisella tularensis*	2	*Streptococcus gordonii*	1
*Haemophilus influenzae*	4	*Streptococcus mitis*	2
*Haemophilus parainfluenzae*	2	*Streptococcus mutans*	1
Human gDNA	2	*Streptococcus oralis*	1
*Klebsiella oxytoca*	1	*Streptococcus pneumoniae*	56
*Klebsiella pneumoniae*	8	*Streptococcus pyogenes*	13
*Lactococcus lactis*	1	*Streptococcus salivarius*	2
*Legionella pneumophila*	1	*Streptococcus thermophilus*	1
*Listeria monocytogenes*	1	*Streptococcus uberis*	1
*Micrococcus sp.*	1	*Streptococcus viridans* grp	8
*Moraxella catarrhalis*	7	Vancomycin Resistant *Enterococcus*	4
*Mycobacterium avium*	1	*Yersinia pestis*	1
*Mycoplasma pneumoniae*	1	*Yersinia pseudotuberculosis*	1
		Total	328

Out of the 328 strains from approximately 80 species, none amplified.

The limit of detection of the Taqman assay was assessed using a dilution series of DNA from isolates of *B. pseudomallei* and *B. mallei*. DNA was quantified using an in-house 16S real-time qPCR assay (unpublished). Template amounts ranging from 10^6^ to 10^0^ genome copies per reaction were used for limit of detection determination.

## Results

Genome comparisons revealed 1,235 SNPs with shared character states among all *B. mallei* genomes that differ from the character state shared by all *B. pseudomallei* genomes. Filtering the 1,235 SNPs using a mismatch value of 100 resulted in a pool of 74 SNPs. The GenBank BLAST search revealed the exclusivity of one of the regions to *B. pseudomallei* and *B. mallei*, so it was selected for assay development and validation.

**Figure 1:**
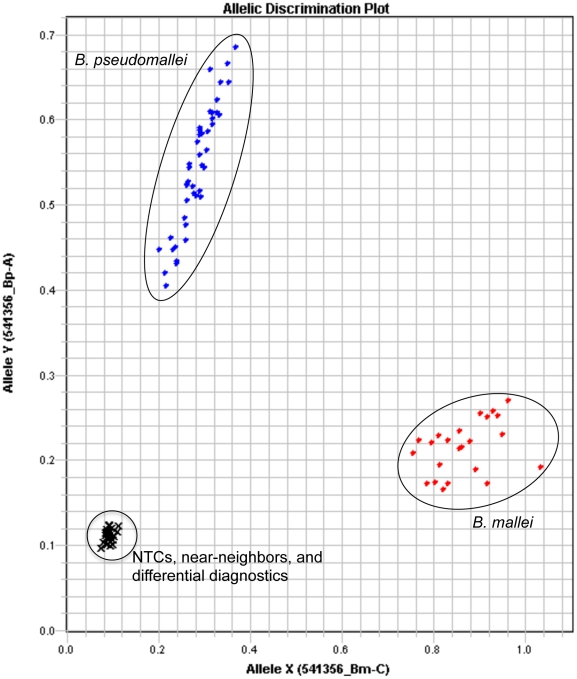
BurkDiff allelic discrimination plot. Results from the assay across 45 *B. pseudomallei* and 23 *B. mallei* strains are shown, along with 2 no template controls (NTCs) and 26 near-neighbor and differential diagnostic species.

**Figure 2:**
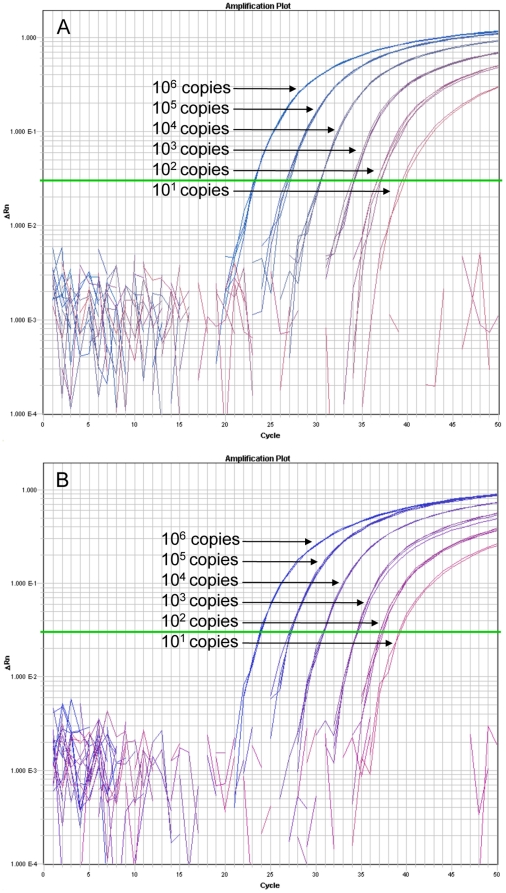
Amplification plots of BurkDiff. Quadruplicates of 10-fold serial dilutions of DNA from a crude heat lysis extraction of A. *B. mallei* strain 2002734303 and B. *B. pseudomallei* strain 2002721637 were screened on BurkDiff to determine the limit of detection of the assay. Two of 4 replicates at the 10^1^ copies template amount did not amplify for both species.

Out of the isolates screened with BurkDiff, all 469 *B. pseudomallei* strains were shown to contain the allele with the SNP state A, and all 49 *B. mallei* strains were shown to contain that with the SNP state C (Table 1, Figure 1). No amplification of DNA from the 390 non-target species was detected, including the *B. oklahomensis* and the *B. thailandensis*-like strain MSMB43, both of which cross-reacted with a previously published allelic discrimination assay [20]. The limit of detection analysis showed consistent detection and allelic discrimination of *B. pseudomallei* and *B. mallei* at DNA template levels as low as 10^2^ genome copies with sporadic amplification and genotyping at <10^2^ genome copies (Figure 2).

## Discussion

The universality of the clinical manifestations of human melioidosis and glanders precludes patient presentation as a definitive diagnostic for these diseases [3], [22]. Diagnosis by traditional methods can be too time-consuming, or require special equipment [1]. The intrinsic resistance of *B. pseudomallei* and *B. mallei* to many widely-used antibiotics and the swift downward progression of untreated or inappropriately-treated *B. pseudomallei*- and *B. mallei*-infected patients necessitate the rapid, specific identification of these species in the clinic [1], [3]. The likelihood of infection with *B. pseudomallei* and *B. mallei* may not be equal given the circumstances; factors such as geographical distribution, prevalence, and risk factors for the diseases would be used in clinical practice for diagnosis. However there are scenarios in which the two species' distinction is necessary. The trade restrictions imposed when animal glanders is diagnosed, but not animal melioidosis, and the potential use of *B. pseudomallei* or *B. mallei* as a bioterrorism agent both underscore the need to differentiate between the two species for reporting purposes and forensic tracking [22], [23]. Rapid species identification may assist with appropriate initial patient treatment for human glanders. Currently physicians prescribe the lengthy drug regimen particular for melioidosis to human glanders patients [3], despite the differing *in vitro* antibiotic susceptibility profiles of the two causative agents [24]. Of the rapid molecular methodologies with the capabilities of identifying and differentiating between *B. pseudomallei* and *B. mallei*, BurkDiff is unique in being single-step, single-reaction. In cases in which circumstances eliminate the possibility of one of the two species, BurkDiff can be used as a single-probe assay for specific identification.

The increasingly cosmopolitan nature of human activity inevitably exposes non-endemic area residents to *B. pseudomallei* and *B. mallei*, increasing the need for heightened awareness of these organisms outside their areas of endemicity, as has been demonstrated by numerous cases of imported melioidosis [25], [26]. Most of these cases can be attributed to exposure during travel to tropical areas. However, global trade in commodities such as animals, plants and soils, and food items is also a possible transmission source resulting in disease in individuals with no travel history [27]. Diagnostic capabilities in non-endemic areas are becoming essential, not only for rapid, appropriate patient treatment, but for the safety of laboratory workers culturing the unknown organism for diagnosis [26]. Molecular assays, including BurkDiff, are rapid, sensitive, and specific, requiring only the appropriate thermal cycler and reagents common to many labs and obviating the need for direct culture of a dangerous pathogen. In fact, BurkDiff was successfully used among a panel of real-time assays targeting *B. pseudomallei* in the confirmation and characterization of a melioidosis case in an Arizona resident with no travel history [27].

Our use of whole genome sequence data allowed for targeted identification of phylogenetically informative markers (*i.e.*, SNPs) to distinguish between *B. pseudomallei* and *B. mallei*, a preferred method to random identification of SNPs in conserved genes, as was done previously [20]. Additionally, *in silico* analyses of the markers allowed for the design of a highly specific assay. The illustrated specificity of BurkDiff to *B. pseudomallei* and *B. mallei* suggests that insertion of the genomic region that this assay targets occurred during or subsequent to the *B. pseudomallei*/*B. mallei* evolutionary split from its close genetic relative *B. thailandensis* [28]. Our data also suggest that the SNP targeted by BurkDiff is from a subsequent point mutation that occurred after the *B. mallei* lineage diverged from *B. pseudomallei* [28]. The number and diversity of the *B. pseudomallei* and *B. mallei* isolates successfully genotyped using BurkDiff suggest the genomic insertion is evolutionarily stable and therefore a good target for identifying the species, while the point mutation could now be considered a canonical SNP (canSNP), a point mutation that marks a point of evolutionary divergence of two taxa and is inherently stable and thus definitive [29].

BurkDiff adds to the growing number of molecular based assays, especially real-time PCR, that have been designed to detect *B. pseudomallei* and/or *B. mallei*. Using several of these assays in combination for definitive identification could be important, as the Burkholderiaceae are highly recombining organisms [15], [19], [30], and as more and more strains are uncovered, the robustness and sensitivity of these assays will be challenged.

## References

[1] Cheng AC, Currie BJ (2005). Melioidosis: epidemiology, pathophysiology, and management.. Clin Microbiol Rev.

[2] White NJ (2003). Melioidosis.. Lancet.

[3] Gilad J (2007). *Burkholderia mallei* and *Burkholderia pseudomallei*: the causative micro-organisms of glanders and melioidosis.. Recent Pat Antiinfect Drug Discov.

[4] Srinivasan A, Kraus CN, DeShazer D, Becker PM, Dick JD (2001). Glanders in a military research microbiologist.. N Engl J Med.

[5] Whitlock GC, Estes DM, Torres AG (2007). Glanders: off to the races with *Burkholderia mallei*.. FEMS Microbiol Lett.

[6] Rotz LD, Khan AS, Lillibridge SR, Ostroff SM, Hughes JM (2002). Public health assessment of potential biological terrorism agents.. Emerg Infect Dis.

[7] Weissert C, Dollenmaier G, Rafeiner P, Riehm J, Schultze D (2009). *Burkholderia pseudomallei* misidentified by automated system.. Emerg Infect Dis.

[8] Brent AJ, Matthews PC, Dance DA, Pitt TL, Handy R (2007). Misdiagnosing melioidosis.. Emerg Infect Dis.

[9] Wuthiekanun V, Desakorn V, Wongsuvan G, Amornchai P, Cheng AC (2005). Rapid immunofluorescence microscopy for diagnosis of melioidosis.. Clin Diagn Lab Immunol.

[10] Wuthiekanun V, Anuntagool N, White NJ, Sirisinha S (2002). Short report: A rapid method for the differentiation of *Burkholderia pseudomallei* and *Burkholderia thailandensis*.. Am J Trop Med Hyg.

[11] Gee JE, Sacchi CT, Glass MB, De BK, Weyant RS (2003). Use of 16S rRNA gene sequencing for rapid identification and differentiation of *Burkholderia pseudomallei* and *B. mallei*.. J Clin Microbiol.

[12] Lee MA, Wang D, Yap EH (2005). Detection and differentiation of *Burkholderia pseudomallei*, *Burkholderia mallei* and *Burkholderia thailandensis* by multiplex PCR.. FEMS Immunol Med Microbiol.

[13] Thibault FM, Valade E, Vidal DR (2004). Identification and discrimination of *Burkholderia pseudomallei*, *B. mallei*, and *B. thailandensis* by real-time PCR targeting type III secretion system genes.. J Clin Microbiol.

[14] Novak RT, Glass MB, Gee JE, Gal D, Mayo MJ (2006). Development and evaluation of a real-time PCR assay targeting the type III secretion system of *Burkholderia pseudomallei*.. J Clin Microbiol.

[15] Tomaso H, Scholz HC, Al Dahouk S, Eickhoff M, Treu TM (2006). Development of a 5′-nuclease real-time PCR assay targeting *fliP* for the rapid identification of *Burkholderia mallei* in clinical samples.. Clin Chem.

[16] Supaprom C, Wang D, Leelayuwat C, Thaewpia W, Susaengrat W (2007). Development of real-time PCR assays and evaluation of their potential use for rapid detection of *Burkholderia pseudomallei* in clinical blood specimens.. J Clin Microbiol.

[17] Chantratita N, Meumann E, Thanwisai A, Limmathurotsakul D, Wuthiekanun V (2008). Loop-mediated isothermal amplification method targeting the TTS1 gene cluster for detection of *Burkholderia pseudomallei* and diagnosis of melioidosis.. J Clin Microbiol.

[18] Holden MTG, Titball RW, Peacock SJ, Cerdeño-Tárraga AM, Atkins T (2004). Genomic plasticity of the causative agent of melioidosis, *Burkholderia pseudomallei*.. Proc Natl Acad Sci.

[19] Nierman WC, DeShazer D, Kim HS, Tettelin H, Nelson KE (2004). Structural flexibility in the *Burkholderia mallei* genome.. Proc Natl Acad Sci.

[20] U'Ren JM, Van Ert MN, Schupp JM, Easterday WR, Simonson TS (2005). Use of a real-time PCR TaqMan assay for rapid identification and differentiation of *Burkholderia pseudomallei* and *Burkholderia mallei*.. J Clin Micobiol.

[21] Pearson T, Giffard P, Beckstrom-Sternberg S, Auerbach R, Hornstra H (2009). Phylogeographic reconstruction of a bacterial species with high levels of lateral gene transfer.. BMC Biology.

[22] US Army Medical Research Institute of Infectious Diseases (2005). USAMRIID's medical management of biological casualties handbook, sixth ed.. http://www.usamriid.army.mil.

[23] Neubauer H, Sprague LD, Joseph M, Tomaso H, Al Dahouk S (2007). Development and clinical evaluation of a PCR assay targeting the metalloprotease gene (*mprA*) of *B. pseudomallei*.. Zoonoses Public Health.

[24] Thibault FM, Hernandez E, Vidal DR, Girardet M, Cavallo JD (2004). Antibiotic susceptibility of 65 isolates of *Burkholderia pseudomallei* and *Burkholderia mallei* to 35 antimicrobial agents.. J Antimicrob Chemother.

[25] Cahn A, Koslowsky B, Nir-Paz R, Temper V, Hiller N (2009). Imported melioidosis, Israel, 2008.. Emerg Infect Dis.

[26] Centers for Disease Control and Prevention (2006). Imported melioidosis - South Florida, 2005.. Morbid Mortal Weekly Rep.

[27] Engelthaler DM, Bowers JR, Schupp JA, Pearson T, Ginther J (2010). Molecular investigations of an autochthonous case of melioidosis in Southern Arizona, USA..

[28] Godoy D, Randle G, Simpson AJ, Aanensen DM, Pitt TL (2003). Multilocus sequence typing and evolutionary relationships among the causative agents of melioidosis and glanders, *Burkholderia pseudomallei* and *Burkholderia mallei*.. J Clin Microbiol.

[29] Keim P, Van Ert MN, Pearson T, Vogler AJ, Huynh LY (2004). Anthrax molecular epidemiology and forensics: using the appropriate marker for different evolutionary scales.. Infect Genet Evol.

[30] Tuanyok A, Leadem BR, Auerbach RK, Beckstrom-Sternberg SM, Beckstrom-Sternberg JS (2008). Genomic islands from five strains of *Burkholderia pseudomallei*.. BMC Genomics.

